# Dengue Virus Infects Primary Human Hair Follicle Dermal Papilla Cells

**DOI:** 10.3389/fcimb.2018.00268

**Published:** 2018-08-21

**Authors:** Kai-Che Wei, Mei-Shu Huang, Tsung-Hsien Chang

**Affiliations:** ^1^Department of Dermatology, Kaohsiung Veterans General Hospital, Kaohsiung, Taiwan; ^2^Faculty of Yuh-Ing Junior College of Health Care and Management, Kaohsiung, Taiwan; ^3^Department of Medical Education and Research, Kaohsiung Veterans General Hospital, Kaohsiung, Taiwan; ^4^Department of Medical Laboratory Science and Biotechnology, Chung Hwa University of Medical Technology, Tainan, Taiwan

**Keywords:** dengue virus, hair loss, human hair follicle dermal papilla cells, cell death, inflammation

## Abstract

During the epidemic of the dengue virus (DENV) infection in Taiwan in 2014 and 2015, we observed an abnormally high frequency of increased scalp hair shedding in infected individuals that could not be explained by telogen effluvium. In this study, the mechanism of hair loss caused by DENV was explored. Human hair follicle dermal papilla cells (HFDPCs) are essential for hair follicle morphogenesis and cycling. Thus, we established an *in vitro* DENV infection model in HFDPCs. On immunofluorescence analysis, HFDPCs that were susceptible to DENV infection responded to type I interferon (IFN) treatment, and the cells showed antibody-dependent enhancement (ADE) effect. The expression of the pro-inflammatory cytokines, interleukin 6 (IL-6), and tumor necrosis factor-alpha (TNF-α), revealed an inflammatory response in DENV-infected HFDPCs. In particular, DENV infection impaired cell viability, and it activated caspase-associated cell death signaling in HFDPCs. In conclusion, our data indicate that direct infection with DENV causes inflammation and cell death in HFDPCs, which is involved in the mechanisms of hair loss after DENV infection. The knowledge of DENV infection in an immune-privileged tissue, such as hair follicles, may suggest their use for further studies on post-dengue fatigue syndrome (PDFS).

## Introduction

South Taiwan experienced an epidemic of dengue virus (DENV) infection in 2014 and 2015, with more than 50,000 confirmed cases of dengue. Dengue virus type 1 (DENV-1) was the main causative agent in 2014, and in 2015 DENV type 2 was the causative agent (Wang et al., 2016). During the epidemic, we observed an abnormally high frequency of increased scalp hair shedding in infected individuals, without scarring or permanent hair loss. This type of hair loss occurred as early as the first month after acute dengue infection. However, the cellular reason for DENV-mediated hair loss was unknown.

Reports of hair loss with DENV infection are sporadic (Harn, 1989; Qiu et al., 1993; Jensenius et al., 1997; Tristão-Sá et al., 2012; Hitani et al., 2015; Chu and Yang, 2017). Our clinical observations agreed with these reports that the hair loss was not associated with the dengue infection severity (Tristão-Sá et al., 2012). Hair loss occurred in many victims as early as within the initial 2 weeks postinfection. More than two-thirds of the cases occurred within the first 2 months postinfection (Qiu et al., 1993; Jensenius et al., 1997; Tristão-Sá et al., 2012; Hitani et al., 2015; Chu and Yang, 2017). The timing of hair shedding, which occurs about 2–3 months after acute infection, could not be fully explained by telogen effluvium. Telogen effluvium is a form of non-scarring alopecia characterized by diffuse hair shedding that is triggered when a physiological stress, such as systemic infection, causes large number of hairs to enter the telogen or the resting phase of the hair follicle at the same time. Massive hair loss is not observed until the new anagen hairs begin to grow. The emerging hairs help to force the resting hairs out of the follicle. Hence, the interval between the inciting event in telogen effluvium and the onset of shedding corresponds to the length of the telogen phase, which is about 3 months. From our experience and based on literature, it could be said that the onset of hair loss associated with DENV infection was too early to be explained well by telogen effluvium.

Previous studies focused on the role of hematopoietic-related cells and endothelium in DENV infection, and DENV has been found to infect, and replicate in, multiple cell types such as hematological lineage cells, liver, and endothelium (Noisakran et al., 2010, 2012). We speculated that DENV might cause hair loss by infecting hair follicles or by interfering with hair growth through infection of hair follicle-associated cells. However, the interaction between DENV and hair follicles has not been evaluated. The human hair follicle is composed of epidermal (epithelial) and dermal (mesenchymal) compartments (Millar, 2002). Human hair follicle dermal papilla cells (HFDPCs) are mesenchymal cells that can be isolated from the hair papilla of normal human scalp hair follicles. Hair papilla in the adult hair follicle plays a crucial role in the dermal-epidermal interactions that control hair production and the events of the hair growth cycle (Driskell et al., 2011). Since the deletion of specific genes in early dermal papilla has revealed a significant signaling impairment during the hair follicle morphogenesis (Ramos et al., 2013), it has been suggested that the dermal papilla is the center for signaling network.

Hence, we used HFDPCs to clarify whether DENV can infect such cells and to study the cellular responses, such as inflammation and cell death, of DENV-mediated hair loss. Antibody-dependent enhancement (ADE) occurs when non-neutralizing antiviral proteins facilitate virus entry into host cells, thereby leading to increased infectivity in cells (Martina et al., 2009). We also assayed for ADE in HFDPCs.

## Materials and methods

### HFDPCs

We used commercial primary HFDPCs, which are mesenchymal cells isolated from the hair papilla of normal human scalp hair follicles (Cat. #602-05a and Cat. #602t-05a, Cell Applications, Inc. San Diego, CA). HFDPCs were cultured in specific growth medium (Cell Applications, Inc.) supplemented with 12% fetal bovine serum (FBS) at 37°C and 5% CO_2_.

### Dengue virus

The strains, DENV-1 (766733A) and DENV-2 (PL046) (GenBank accession no. AJ968413.1), were isolated from patients with dengue fever and were kindly provided by Yi-ling Lin (Academia Sinica, Taipei) (Lin et al., 1998). They were propagated in the mosquito cell line C6/36 (ATCC: CRL-1660) grown in RPMI 1640 medium containing 5% FBS. To determine virus titers, culture medium from DENV-2 infected C6/36 cells was harvested for plaque-forming assay. Various virus dilutions were added to 80% confluent baby hamster kidney (BHK-21) cells (BCRC: 60041, Hsinchu, Taiwan) and were incubated at 37°C for 2 h. After adsorption, cells were washed and overlaid with 1% agarose (SeaPlaque; FMC BioProducts, Philadelphia, PA) containing RPMI 1640 medium with 1% FBS. After 7 days of incubation, cells were fixed with 10% formaldehyde and stained with 0.5% crystal violet.

### Viral infection

HFDPCs (4 × 10^4^ cells/well in 12-well plates) were replaced with serum free medium, and then infected or not infected (untreated control) with DENV at a multiplicity of infection (MOI) of 10 or 50. In some experiments, DENV-2 with an MOI of 0.1–10 was used. After 4 h of adsorption, the virus soup was removed, and the cells were incubated with growth medium supplemented with 2% FBS for 4 days. In some cases, HFDPCs were pretreated or not pretreated (untreated control) with 500 IU/ml of recombinant human interferon alpha-2a (IFNα-2a, Cat. #Cyt-204, ProSpec-Tany TechnoGene Ltd, Ness-Ziona, Israel) for 16 h, and then the cells were infected with DENV-2. For ADE assay, HFDPCs were incubated with or without (untreated control) anti-envelope (E) protein antibody (α-E, mouse monoclonal antibody, #YH0025, 1:10,000, Yao-Hong Biotechnology, Taipei) and DENV-2 (MOI = 10 or 50).

### Immunofluorescence assay

Human hair follicle dermal papilla cells were fixed with 4% paraformaldehyde for 30 min, and then permeabilized with 0.5% Triton X-100 for 10 min. After two washes with phosphate buffered saline (PBS), cells were blocked with 10% skim milk in PBS. Infected cells were detected by incubation with the antibody targeting NS3 (#YH0034, 1:10,000, Yao-Hong Biotechnology), and then with the secondary anti-mouse Alexa Fluor 488 antibody (Thermo Fisher Scientific, Waltham, MA) (Wang et al., 2015). DAPI was used to stain nuclei. Fluorescence signals were observed by fluorescence microscopy (Zeiss, Axio Observer A1, Oberkochen Germany).

### Cell viability assay

The WST-1 assay (Roche, Basel, Switzerland) was used to assess cell viability. HFDPCs were grown at a density of 5 × 10^3^ cells/well in 96-well plates overnight, followed by infection with DENV (MOI = 10, MOI = 50). After 4 days of incubation, 10 μl of Cell Proliferation Reagent WST-1 was added directly to each well, and then was incubated again for 2 h at 37°C. Cell viability was quantified by multi-well spectrophotometry (Anthos, Biochrom, Cambridge, UK). Absorbance at 450 nm was tested, and the reference wavelength was set at 620 nm. The absorbance was related to the cell viability percentage using the formula [(OD of infection test/OD of untreated control) × 100].

### Western blot analysis

Cells were lysed in RIPA buffer (150 mM NaCl, 0.5% sodium deoxycholate, 1% NP40, 0.1% SDS, 50 mM Tris-HCl [pH 8.0]) or SDS buffer (2% SDS, 50 mM Tris-HCl [pH 7.5]) containing protease inhibitor and phosphatase inhibitor cocktail (Roche). Harvested extracts were separated by 10% SDS-PAGE and transferred to PVDF membranes. Later, the membranes were incubated with primary antibody, and then with horseradish peroxidase-conjugated secondary antibody (Jackson ImmunoResearch Laboratory, West Grove, PA) and were finally visualized using enhanced chemiluminescence (Thermo Fisher Scientific). Image acquisition and signal density measurements were carried out using the BioSpectrum Image System (UVP, Upland, CA). The following primary antibodies were used: anti-caspase 1 (GTX111630, GeneTex, Irvine, CA), anti-caspase 3 (#9665, Cell Signaling Technology, Danvers, MA), anti-caspase 7 (GTX1002337, GeneTex), anti-caspase 8 (#4790, Cell Signaling Technology), anti-bone morphogenetic protein 4 (BMP-4; GTX100875, GeneTex), anti-phospho-STAT1 (phospho-Tyr701, #9167 Cell signaling), anti-STAT1 (#14994, Cell Signaling), anti-phospho-STAT2 (phospho-Tyr690, GTX50721, GeneTex), anti-STAT2 (#14994, Cell Signaling), anti-DENV NS3 (GTX124252, GeneTex), and anti-GAPDH (#60004-1-Ig, Proteintech Group, Rosemont, IL).

### RT^2^ profiler PCR array and RT-qPCR

Total RNA was extracted from HFDPCs by using Trizol reagent (Thermo Fisher Scientific) as instructed in the protocol. Total RNA (1,000 ng) from each sample underwent cDNA synthesis by using the RT^2^ First Strand Kit (Qiagen, Valencia, CA). The resulting cDNA was mixed with H_2_O plus SYBR green dye, and then dispensed into a 96-well RT^2^ Profiler PCR array plate as described (Qiagen). DNA amplification was carried out using the Applied Biosystems StepONE Plus Real-Time PCR system (Thermo Fisher Scientific). The fold-change in gene expression in DENV-infected HFDPCs was relative to that of the untreated control. We considered a fold-change threshold of at least two-fold upregulation or downregulation.

The specific gene expression was also confirmed by RT-qPCR. cDNA was synthesized from 500 ng total RNA using Superscript III Reverse Transcriptase (Invitrogen). qPCR amplification was carried out with 3 ng of cDNA in 10 μl SYBR Green using the Applied Biosystems StepONE Plus Real-Time PCR system (Thermo Fisher Scientific). Transcript levels were normalized to that of glyceraldehyde 3-phosphate dehydrogenase (GAPDH). The primer sequences for gene detection are provided in Table S1.

### Statistical analysis

Significant differences between the groups were determined by Student's *t-*test using GraphPad Prism (La Jolla, CA). Data are expressed as mean ± SD from 3~4 independent experiments. *P* < 0.05 was considered to be statistically significant.

## Results

### DENV-1 and DENV-2 infection of HFDPCs

**Figure 1:**
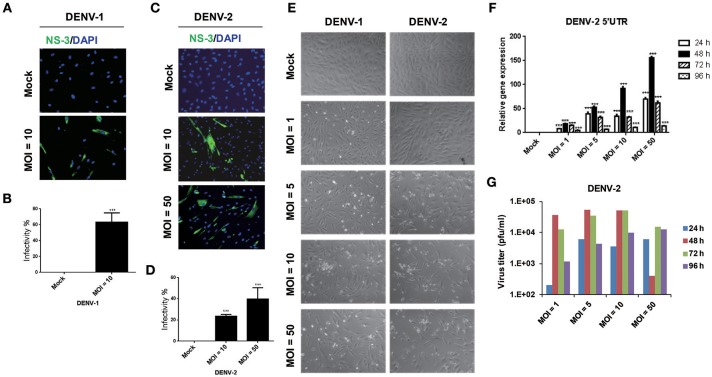
Infective ability of dengue virus type 1 (DENV-1) and DENV-2 in hair follicle dermal papilla cells (HFDPCs). **(A,C)** Immunofluorescence assay of HFDPCs with DENV-1 (MOI = 10) and DENV-2 (MOI = 10 and 50) infection for 4 days. Shows NS3 protein signal (green fluorescence) of DENV infection and DAPI staining (blue fluorescence) for cell nuclei. **(B,D)** Quantification of the DENV-1 and DENV-2 infectivity in HFDPCs. Data are mean ± SD of three observation fields. ***P < 0.005 vs. untreated control. **(E)** The cell morphology of HFDPCs with DENV-2 infection (MOI = 1, 5, 10, and 50) for 72 h under brightfield microscope were observed and captured. **(F)** RT-qPCR of DENV-2 5′-UTR expression in HFDPCs infected with DENV-2 (MOI = 1, 5, 10, and 50) were performed at 24, 48, 72, and 96 h postinfection. The gene expression was normalized to GAPDH gene. Data are mean ± SD from three independent tests, ***P < 0.005 vs. untreated control. **(G)** Detection of DENV-2 virions in the growth medium of HFDPCs. The growth medium of HFDPCs with DENV infection (MOI = 1, 5, 10, and 50) were harvested at 24, 48, 72, and 96 h postinfection by virus titration plaque assay in BHK-21 cells.

During the dengue outbreak in Taiwan in 2014 and 2015, DENV-1 and DENV-2 were the most prevalent serotypes (Wang et al., 2016); therefore, we used these two serotypes in this study. Since HFDPCs are indispensable for regenerating new hair follicles, we investigated whether HFDPCs were susceptible to DENV infection. HFDPCs were infected with DENV-1 (MOI = 10) and DENV-2 (MOI = 10 and 50). After 4 days, DENV-infected cells were detected by immunofluorescence assay; the infectivity of DENV-1 was 63% (MOI = 10) (Figures 1A,B) and that of DENV-2 was 23 and 40% (MOI = 10 and 50), respectively (Figures 1C,D). Thus, HFDPCs were susceptible to infection with DENV, particularly DENV-1. Compared with the untreated infection control, the morphology of HFDPCs infected with DENV-1 and DENV-2 changed, and the cytopathic effect (CPE) was also observed (Figure 1E). Furthermore, the DENV-2 5′-untranslated region (UTR) gene replication was detected in HFDPCs with DENV-2 infection (MOI = 1, 5, 10, and 50). The viral RNA replication peak was detected at 48 h post-infection, but the peak decreased at 72 and 96 h, which suggested that the severe CPE could not support the replication of DENV in HFDPCs (Figure 1F). The virions were also detected in the culture medium of DENV-2-infected HFDPCs, and the titration assay showed a similar result with viral RNA detection, which indicated that DENV replicated in HFDPCs without CPE (Figure 1G).

### IFNα attenuates DENV-2 infectivity in HFDPCs

**Figure 2:**
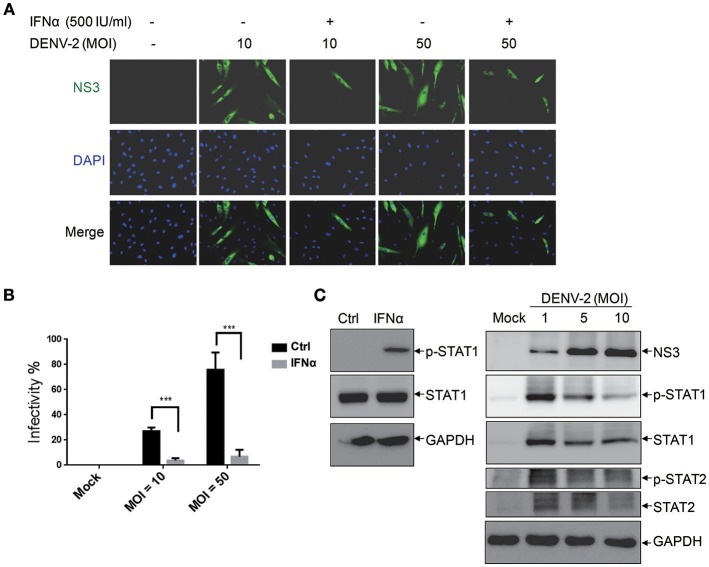
Interferon α (IFNα) inhibits DENV-2 in HFDPCs. **(A)** HFDPCs (4 × 10^4^ cells/well) were pretreated with IFNα (500 U/ml) before DENV-2 infection at MOI 10 and 50 for 4 days. Immunofluorescence assay of DENV-infected cells (green fluorescence) with anti-NS3 antibody. Cell nuclei are stained with DAPI (blue fluorescence). Merged images are also presented. **(B)** Quantification of DENV-2-infected cells. Data are mean ± SD of three observation fields. ***P < 0.005 vs. untreated control. **(C)** Left panels, HFDPCs (1 × 10^5^) were treated with IFNα-2a (1,000 U/ml) for 6 h, the untreated cells are indicated as ctrl. Right panels, HFDPCs infected with untreated control or DENV2 at MOI = 1, 5, and 10 for 4 days. Immunoblotting of levels of phospho-STAT1/STAT2 and total STAT1/STAT2 in whole cell extracts. DENV-2 NS3 indicates viral infection and GAPDH is the loading control. The represented data are from the consistent results of two or three independent experiments.

The activation of the innate immune pathway and inflammatory pathway during dengue disease was revealed, which show a dual role in mediating both protection and exacerbation of disease (Costa et al., 2013). Type I IFN is an antiviral cytokine used against virus infection in host cells, our previous report showed that DENV-2 was able to induce IFNβ production in cells (Chang et al., 2006); however, its antiviral activity could be modulated by DENV (Yu et al., 2012). Thus, it would be important to understand whether the antiviral activity of IFNα is effective against DENV infection in HFDPCs. The cells were pretreated with IFNα before infection with DENV-2 (MOI = 10 and 50). Interferon-alpha treatment significantly decreased the DENV-2 infectivity from 27 to 3% at MOI = 10 and from 76 to 7% at MOI = 50 as compared with untreated control cells (Figures 2A,B). These data provided evidence for IFNα mediated inhibition of DENV-2 infection in HFDPCs.

We revealed the effect of type I IFN against DENV-2-infected HFDPCs (Figures 2A,B). Therefore, we measured the level of Janus kinase (JAK)-signal transducer and activator of transcription 1 and 2 (STAT1 and STAT2) activation in the type I IFN signaling pathway. The studies showed that IFNα-2a induced the expression of phosphorylated STAT1 in HPDPCs (Figure 2C, left panels). These data could explain that the pretreatment with type I IFN inhibited DENV-2 infection in HFDPCs. The immunoblots showed that the high level of STAT1/2 phosphorylation was induced by DENV-2 infection at MOI = 1, but it was decreased by DENV-2 infection at MOI = 5 and 10 in HPDPCs (Figure 2C, right panels), which indicated that a large amount of DENV-2 infection was able to downregulate the IFN signaling pathway.

### ADE increases the infective ability of DENV-2 in HFDPCs

**Figure 3:**
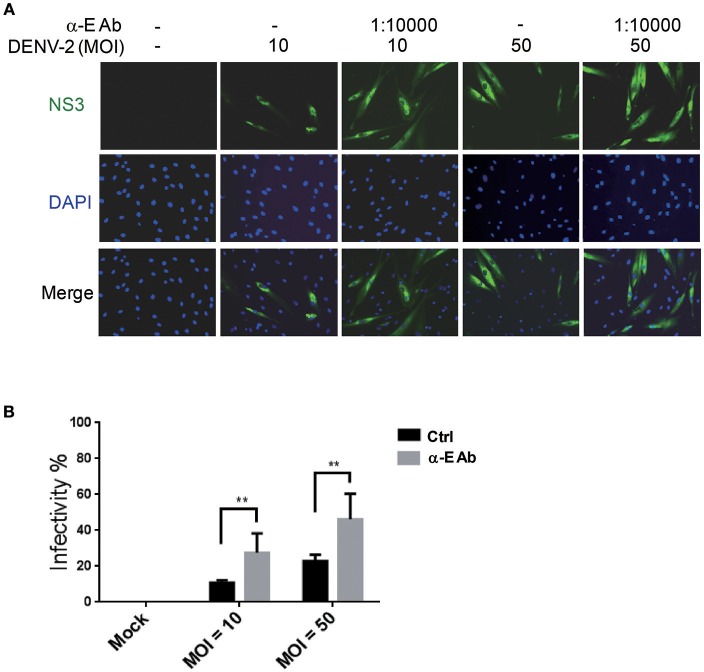
Antibody-dependent enhancement increases DENV-2 infectivity in HFDPCs. **(A)** HFDPCs (4 × 10^4^ cells/well) were infected with DENV-2 (MOI = 10 and 50) with α-E antibody (10,000-fold dilution). After 4 days of infection, HFDPCs were stained with anti-NS3 antibody and DAPI to detect DENV (green color) and cell nuclei (blue color), respectively. **(B)** The infectivity rate was quantified. Data are mean ± SD of three observation fields. **P < 0.01 vs. untreated control.

Subneutralizing levels of antibodies have been shown to enhance DEN-V replication *in vitro* and disease severity in animal models (Zellweger et al., 2010). In addition, the ADE is also directly associated with the severity of secondary dengue disease in humans (Katzelnick et al., 2017). This is because ADE can enhance the entry of DEN-V into hematopoietic cells (Flipse et al., 2016b). We would like to understand whether the ADE effect accelerates the infection of DENV-2 in HFDPCs. Previous studies have demonstrated that the Fc receptors are functionally expressed in human keratinocytes and skin-derived human mast cells (Cauza et al., 2005; Zhao et al., 2006). Thus, we measured the FcγR mRNA expression in HFDPCs and in the control cells, HaCaT keratinocytes. When compared with HaCaT cells, HFDPCs expressed a higher mRNA level of Fc receptors, such as FcγRIIA, FcγIIB, and neonatal Fc receptor (FcRn) (Figure S1A). The FcRIIA/B protein expression in the surface of HFDPCs was also detected (Figure S1B). Then, we added anti-E protein antibody to cultures during virus adsorption. This treatment significantly increased DENV-2 infectivity from 11 to 28% at MOI = 10 and 23 to 46% at MOI = 50 as compared with untreated control cells (Figures 3A,B). These data suggest that ADE might play roles in the pathogenic infection of HFDPCs.

### DENV induces inflammatory cytokine expression in HFDPCs

**Figure 4:**
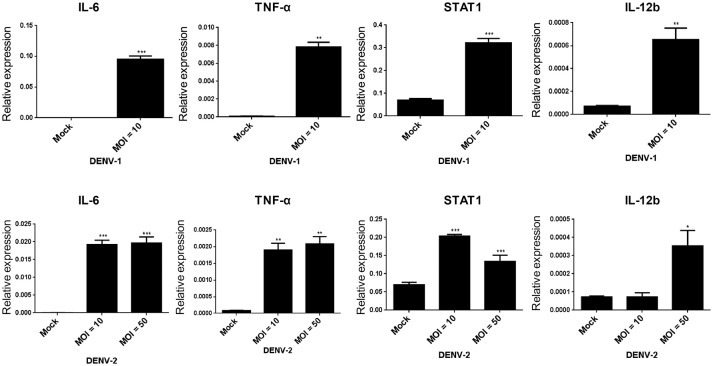
DENV-1 and−2 induce interleukin 6 (IL-6), tumor necrosis factor-alpha (TNF-α), signal transducer, and activator of transcription 1 (STAT1), and IL-12b gene expression in HFDPCs. RT-qPCR of IL-6, TNF-α, STAT1, and IL-12b expression in HFDPCs infected with DENV-1 (MOI = 10) and DENV-2 (MOI = 10 and 50) for 4 days. The gene expression was normalized to GAPDH gene. Data are mean ± SD from three independent tests, *P < 0.05; **P < 0.01; ***P < 0.005 vs. untreated control.

On DENV infection, an appropriate inflammatory response would be activated in the host to clear the pathogen, thereby limiting the risk of disease. However, the inflammatory activity is also involved in the pathogenesis of DENV (Chaturvedi et al., 2000; Costa et al., 2013). The NF-κB activity mediated inflammatory response was evaluated in HFDPCs with lipopolysaccharide (LPS) challenge (Hill et al., 2012). So, it is important to understand whether DENV triggers pro-inflammatory cytokine expression in HFDPCs. Our data showed that DENV-1 and DENV-2 induced the expression of the pro-inflammatory cytokines interleukin 6 (IL-6), tumor necrosis factor-alpha (TNF-α), and IL-12b, as well as the downstream mediator STAT1 in HFDPCs (Figure 4). In addition, IL-1β, IL-6, and IL-8 expression and the DENV-2 5′-UTR region were induced by various MOI (0.1–10) of DENV-2 (Figure S2). Therefore, DENV infection triggered an inflammatory response in HFDPCs.

### DENV impairs the cell viability of HFDPC

**Figure 5:**
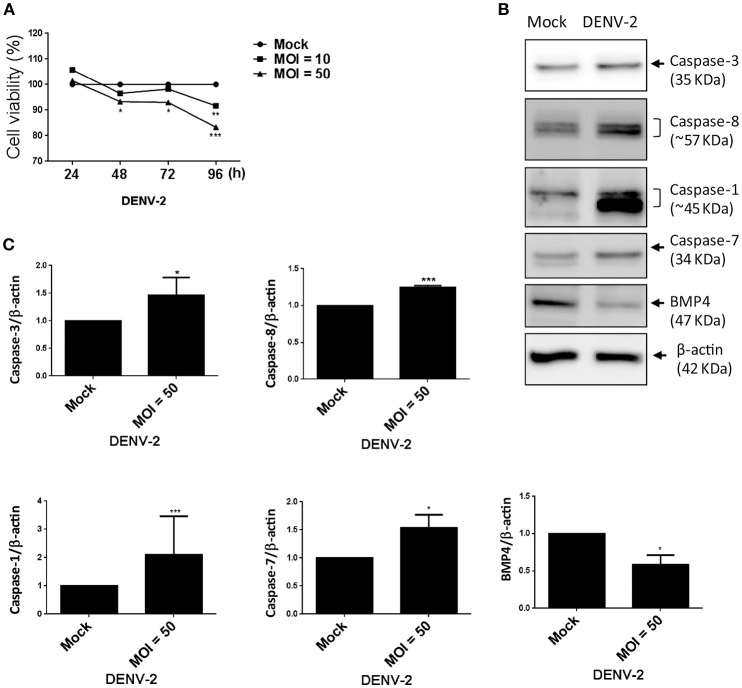
DENV-2 induces cell-damage and apoptosis pathway in HFDPCs. **(A)** HFDPCs (4 × 10^4^ cells/well) were infected with DENV-2 (MOI = 10 and 50), and cell viability was detected by WST-1 assay at 24, 48, 72, and 96 h postinfection. Data are mean ± SD from three independent tests. *P < 0.05, **P < 0.01, ***P < 0.001 vs. untreated control. **(B)** Western blot analysis of protein expression of caspase 3, caspase 8, caspase 1, and caspase 7, and BMP-4 in HFDPCs (2 × 10^5^ cells/well) infected with DENV-2 (MOI = 50) for 4 days. β-actin expression was an internal loading control. The full membrane images are shown in Figure S4. **(C)** Quantification of protein levels (mean ± SD) from three independent assays. *P < 0.05 and ***P < 0.005 vs. untreated control.

Viral infection-mediated cell death is a strategy of host to restrict virus replication and spread (Okamoto et al., 2017). In Figure 1E, we observed the CPE in HFDPCs and detected lower levels of DENV-2 RNA and virions at late infection phase (Figures 1F,G). Again, by using the WST-1 cell proliferation assay, we detected that DENV-2 significantly decreased the cell viability after DENV-2 infection for 96 h (Figure 5A). Also, the levels of caspase 3, caspase 7, and caspase 8, and factors of apoptosis were increased in DENV-2-infected HFDPCs (Figures 5B,C and Figure S3), which suggested that the apoptosis-associated caspase cascade was activated by DENV-2. Bone morphogenetic protein signaling in HFDPCs is required for the growth and differentiation of hair follicles (Rendl et al., 2008; Solanas and Benitah, 2013). The protein level of BMP-4 was reduced with DENV-2 infection (Figures 5B,C). We also found that, DENV downregulated the level of dermal papilla signature genes, such as alkaline phosphatase (ALPL), noggin (NOG), lymphoid enhancer-binding factor 1 (LEF-1), and wingless-related MMTV integration sit 5A (WNT5A), in HFDPCs (Figure S4); these molecules are required in the maintenance of intrinsic dermal papilla properties (Ohyama et al., 2012). These data indicated a dysfunction of supporting hair growth in DENV-2-infected HFDPCs.

We used RT^2^ Profiler PCR Array to assay the DENV-triggered cell death signaling pathways in HFDPCs, including genes in the pathways of autophagy (cathepsin S, FAS, and TNF), apoptosis (caspase 1, CD40, C-abl oncogene 1, and caspase 7), and necrosis (forkhead box I1). The genes' altered expression was shown in the scatter plot (Figure S5) and confirmed by RT-qPCR (Table 1) and suggested that DENV-2 infection activated cell damage/death signaling in HFDPCs.

**Table 1:** Changes in gene expression in dengue virus type 2 (DENV-2)-infected hair-follicle dermal papilla cells (HFDPCs).

**Pathway**	**Gene**	**PCR array**	**qRT-PCR**	***P*-value qRT-PCR**
Autophagy	CTSS	404.06	131.78 ± 92.5	<0.001
	FAS	2.16	1.36 ± 0.26	<0.001
	TNF	2.68	2.21 ± 0.86	<0.001
Apoptosis	CASP1	54.90	31.72 ± 21.72	<0.001
	CD40	6.02	1.58 ± 0.41	<0.001
	ABL1	2.11	2.16 ± 0.82	<0.001
	CASP7	2.05	1.41 ± 0.30	<0.001
Necrosis	FOXI1	2.62	2.32 ± 0.94	<0.001

*Data are represented as fold change (DENV-2 treated vs. untreated control). CTSS, cathepsin S; TNF, tumor necrosis factor; CASP1 and 7, caspase 1 and 7; ABL1, C-abl oncogene 1; FOXI1, forkhead box I1*.

## Discussion

In this study, we demonstrated that HDFPCs were susceptible to DENV infection, which could be related with the massive scalp hair loss observed with the epidemic of DENV infection in Taiwan in 2014 and 2015. The infection could be reduced by type I IFN treatment. The ADE effect, frequently seen in macrophages (Flipse et al., 2016a), was also found in HFDPCs. The expression of the pro-inflammatory cytokines IL-6 and TNF-α implied that there is an inflammatory response in HFDPCs after DENV infection. In particular, DENV caused cell death, activated caspase-associated cell death signaling, and reduced BMP-4 protein level. Direct DENV infection of HFDPCs might play roles in the post-dengue fatigue syndrome (PDFS), including hair loss. Our findings provide further evidences about the pathophysiology of hair loss after DENV infection.

Our data show that primary non-immortalized HFDPCs could be infected with DENV-1 and DENV-2 with various MOI of inoculation dose; this is the first evidence of DENV targeting HFDPCs (Figure 1). However, the virus infectivity was comparatively poorer with non-immortalized HFDPCs than with other immortalized cell lines we reported earlier, such as A549 lung carcinoma cells or WS1 skin fibroblastomas, in which effective DENV infection could be achieved at MOI = 1 with an infection period of 24 h (Wang et al., 2015). Since the information on DENV tissue tropism is limited, DENV localization was never detected in scalp tissue (Jessie et al., 2004; Balsitis et al., 2009). Thus, the discrepancy of DENV infectivity between HFDPCs and other cell types remains to be further explored.

Antibody-dependent enhancement is important in helping the virus invade hematopoietic cells and it plays critical roles in the pathophysiology of dengue hemorrhagic fever (Flipse et al., 2016b). The non-neutralizing antibody can help DENV to enter human hematopoietic lineage cells, because DENV-antibody complexes are targeted to Fcγ receptor (FcγR)-bearing cells (Flipse et al., 2016b). Upon interaction of the antibodies with FcγR, the virion is internalized in the cell (Martina et al., 2009). The polymorphism of Fc gamma receptors (FcγR) was found to be a genetic factor involved in the development of dengue disease (Loke et al., 2002). We evaluated the expression of different FcγR in HFDPCs, the results showed that the FcγRIIA expression level was higher than the other types of FcγR (Figure S1). Interestingly, ectopic expression of FcγRIIA (CD32) was found to enhance DENV immune complex infectivity more effectively than FcγRIA (CD64) in COS-7 cells (Rodrigo et al., 2006), this report may partly support our ADE analysis in HFDPCs. Since ADE has never been reported in hair follicle-associated tissue, ours is the first study which revealed that ADE facilitates the infectivity of DENV in HFDPCs (Figure 3), which suggests that the serotype cross-reactive antibody might be involved in hair loss with PDFS. However, it remains unclear whether secondary or more severe DENV infections cause higher frequency or degree of hair loss in patients. Further investigations would be needed to answer this issue.

Type I IFN plays a key role against virus infection in hosts (Chang et al., 2006; De La Cruz Hernandez et al., 2014; Palma-Ocampo et al., 2015), this study supports our finding that type I IFN efficiently inhibits DENV replication in HFDPCs (Figures 2A,B). We also found that STAT1/2 was highly activated in HFDPCs with low MOI of DENV-2 infection (MOI = 1), but this high level of STAT1/2 phosphorylation was decreased in cells with high MOI of virus infection (MOI = 5 and 10) (Figure 2C, right panels). The high MOI of DENV-2 infection might feature a type I IFN evasion machinery, which was described in other RNA virus infection models (Lin et al., 2004; Ho et al., 2005; Lu et al., 2012). Dengue virus-induced inflammatory cytokines such as TNF-α, IL-6, and IL-8 are critical in the pathogenesis of dengue fever and dengue hemorrhagic fever (Chaturvedi et al., 2000; Martina et al., 2009). We detected DENV-induced inflammation in HFDPCs as well (Figure 4). The hair follicle constitutes an area of immune privilege, lacking adaptive immunity (Christoph et al., 2000). Thus, inflammatory or cytokine expression in innate immunity might be an important response to invasive microbes or physical stress (Hill et al., 2012; Shin et al., 2016).

We showed that DENV infection induced HFDPC death, caspase cascade activation, and death-associated gene expression, which indicates pathogenic infection in HFDPCs (Figure 5). Dengue virus-caused hair loss may occur by direct infection or by killing hair follicle-associated cells. Furthermore, DENV may interfere with the normal orchestration of the hair cycle through mediators such as cytokines. The hair cycle is tightly controlled by a complex cross talk between hair follicles and the surrounding tissues (Driskell et al., 2011; Higgins et al., 2013). More specifically, hair growth is regulated by the epithelial-mesenchymal interaction of hair follicle cells and the adjacent tissue (dermal papilla and subcutaneous cell tissue), which involves various molecular signaling pathways, including BMP, the Wnt family, fibroblast growth factor, transforming growth factor β, and Hedgehog pathways (Yang and Cotsarelis, 2010). Most of these signals are not derived from hair follicle cells but from adjacent cells. Hair dermal papilla cells maintain bulge stem cells and germ cells in the quiescent state during telogen by producing BMP-4 and interfering with the balance of BMP-4 and BMP inhibitors and fibroblast growth factor (FGF) (Rendl et al., 2008; Solanas and Benitah, 2013). Our data confirms that DENV-infected dermal papilla cells exert an altered signal for hair cycle control by downregulating BMP-4 (Figure 5).

Not only BMP family (Rendl et al., 2008), but also Wnt/β-catenin and FGF have been known to play important roles in affecting HFPCs to regulate hair cycle (Kwack et al., 2013). Furthermore, there are many cellular factors reported to be involved in altering hair cycle, such as ALPL, NOG, LEF-1, and WNT5A (Ohyama et al., 2012; Higgins et al., 2013). Our supplementary data also showed the reduction of ALPL, NOG, LEF-1, and WNT5A genes in DENV-infected HFDPCs (Figure S4). This data suggests that DENV-mediated pathological hair shedding might be through modulation of the signaling activity in hair cycle.

Evidences of DENV infection in an immune-privileged tissue, such as hair follicles, would help to elucidate another clinical puzzle—the PDFS. Indeed, about one-quarter of dengue victims have prolonged chronic fatigue, major depression, and arthralgia for months after recovery, usually 3–6 months, or even up to 2 years (Seet et al., 2007; García et al., 2011; Shin et al., 2016). The mechanisms are still unknown, but autoimmune reaction (García et al., 2011) and continuous virus replication are the possible explanations. Our findings reinforce the possibility that PDFS is caused by the prolonged residence of virus in some immune-privileged tissues, such as hair follicles, where the immune system does not clear the virus; thus, there is still continuous viral shedding after the acute infection phase.

The hair follicle is a highly complex appendage of the skin containing multiple cell types. The follicle undergoes constant cycling throughout the life of the organism, including growth and resorption, with growth being dependent on different cell types. Although HFDPCs play a pivotal role in hair follicle morphogenesis and cycling, the effect of DENV infection in other cell types involved in hair growth should be further studied.

In conclusion, our study confirms that DENV can directly infect hair follicle-associated cells, causing molecular and cytopathic changes and leading to a disturbed hair cycle. Recognizing that DENV can infect hair follicles helps elucidate the pathophysiology of hair loss after DENV infection. Knowledge of DENV infection of immune-privileged tissues, such as hair follicles, may imply the need for further studies to identify their role in PDFS.

## Author contributions

K-CW and T-HC conceived and designed the experiments, analyzed the data, contributed reagents, materials, analysis tools, and wrote the manuscript. M-SH performed the experiments and prepared the figures. All authors reviewed the manuscript.

### Conflict of interest statement

The authors declare that the research was conducted in the absence of any commercial or financial relationships that could be construed as a potential conflict of interest.

## Supplementary material

Table S1 qPCR primer sequences used in this study.

Figure S1 The expression of human Fc gamma receptors in HFDPCs.

Figure S2 Induction of IL-1β, IL-6, and IL-8 expression and the DENV-2 5′-UTR by various MOI (0.1–10) of DENV-2.

Figure S3 The full images of western blotting.

Figure S4 Reduction of ALPL, LEF1, NOG, and WNT5A expression by DENV-1 and DENV-2.

Figure S5 RT^2^ profiler PCR Array analysis of DENV-triggered cell-death signaling pathway.
