# Supplementary material for: Dengue Virus Infects Primary Human Hair Follicle Dermal Papilla Cells
Source: Front Cell Infect Microbiol. 2018 Aug 21;8:268. doi: 10.3389/fcimb.2018.00268 (PMC6110916; doi:10.3389/fcimb.2018.00268)
Supplement: Table S1 — qPCR primer sequences used in this study. [file Data_Sheet_1.PDF]

Supplementary information

Dengue virus infects primary human hair-follicle dermal papilla cells: the implication for dengue-related hair loss

Kai-Che Wei[1,2], Mei-Sui Huang[3], and Tsung-Hsien Chang[3,4*]

[1] Department of Dermatology, Kaohsiung Veterans General Hospital, Taiwan.

[2] Faculty of Yuhing Junior College of Health Care and Management, Kaohsiung, Taiwan

[3]Department of Medical Education and Research, Kaohsiung Veterans General Hospital, Taiwan

[4] Department of Medical Laboratory Science and Biotechnology, Chung Hwa University of Medical Technology, Tainan, Taiwan

Table S1. The sequences of RT-qPCR primers.

**Genes**	**Primer sequences**
human CTSS	F: 5’-ACGACGTCTCATCTGGGAAA-3’<br>R: 5’-GGGAACTCTCAGGGAACTCA-3’
human FAS	F: 5’-ACTGTGACCCTTGCACCAAA-3’<br>R: 5’-CCTTTCTGTGCTTTCTGCATGT-3’
human TNF	F: 5’-CCTCTCTGCCATCAAGAGCC-3’<br>R: 5’-CCTCACAGGGCAATGATCCC-3’
human CASP1	F: 5’-ATCCGTTCCATGGGTGAAGG-3’<br>R: 5’-GCCCCTTTCGGAATAACGGA-3’

human CD40	F: 5’-AGACTGATGTTGTCTGTGGTCC-3’<br>R: 5’-GGCCACCTTTTTGATAAAGACCAG-3’
human ABL1	F: 5’-TCCTCGTCCTCCAGCTGTTA-3’<br>R: 5’-GCAACGAAAAGGTTGGGGTC-3’
human CASP7	F: 5’-GTGGGAACGATGGCAGATGA-3’<br>R: 5’-GAGGGACGGTACAAACGAGG-3’
human FOXI1	F: 5’-CTCTCCACTGCCTTCTGTCC-3’<br>R: 5’-TGTCTGCAGGTAAGTTGGGG-3’
human IL-12β	F-: 5’TGGAGATGCTGGCCAGTACA-3’<br>R-: 5’GCAGGAGCGAATGGCTTAGA-3’
human IL-1β	F-: 5’ACGATGCACCTGTACGATCACT-3’<br>R-: 5’CACCAAGCTTTTTTGCTGTGAGT-3’
human STAT1	F-: 5’GTGGAAAGACAGCCCTGCA-3’<br>R-: 5’ACTGGACCCCTGTCTTCAA-3’
human IL-8	F-: 5’ACACTGCGCCAACACAGAAATTA-3’<br>R-: 5’TTTGCTTGAAG TTCACTGGCATC-3’
human TNFα	F-: 5’GACAAGCCTGTAGCCCATGTTGTA-3’<br>R-: 5’CAGCCTTGGCCCTTGAAGA-3’
human IL-6	F-: 5’TGTCCTGCAGCCACTGGTTC-3’<br>R-: 5’AAGCCAGAGCTGTGCAGATGAGTA-3’
human-ALPL	F-: 5’ATTGACCACGGGCACCAT-3’<br>R-: 5’CTCCACCGCCTCATGCA-3’
human-NOG	F-: 5’GAAGCAGCGCCTAAGCAAGA-3’<br>R-: 5’TGCGACCACAGCCACATCT-3’
human-LEF1	F-: 5’CAGGAGCCCTACCACGACAA-3’<br>R-: 5’CCTCCATCTGGATGCTTTCC-3’

human-WNT5A	F: 5’-CTTGGTGGTCGCTAGGTATGAAT-3’<br>R: 5’-ATGCCCTCCGACGTCTTG-3’
human DENV-2 5' UTR	F: 5’-AGT TGT TAG TCT ACG TGG ACC GA-3’<br>R: 5’-CGC GTT TCA GCA TAT TGA AAG-3’
human GAPDH	F: 5’- TGCACCACCAACTGCTTAGC-3’<br>R: 5’- GGCATGGACTGTGGTCAT-3’
human FcGR2A(CD32)	F:5’-CCAGAATGGAAAATCCCAGAAA-3’<br>R:5’-TTTGCTTGTGGGATGGAGAAG-3'
human FcGR2B(CD32)	F: 5’-TCCAAGCTCCCAGCTCTTCA-3’<br>R:5’-TGCAGTAGATCAAGGCCACTACA-3’
human FcGR3A(CD16a)	F: 5’-AAGACAGCGGCTCCTACTTCTG-3’<br>R:5’-GTTCACAGTCTCTGAAGACACATTTTT-3’
human FcGR3B(CD16b)	F: 5’-GGGCTCCGGATATCTTTGGT-3’<br>F : 5’-AGGAGCAGCTGCCACATGA-3’
human FcRn(neonatal)	F:5’-TCGTGGTGGGAATCGTCATC-3’<br>R:5’-CACGAAGGGAGATCCAAGGG-3’

Figure S1

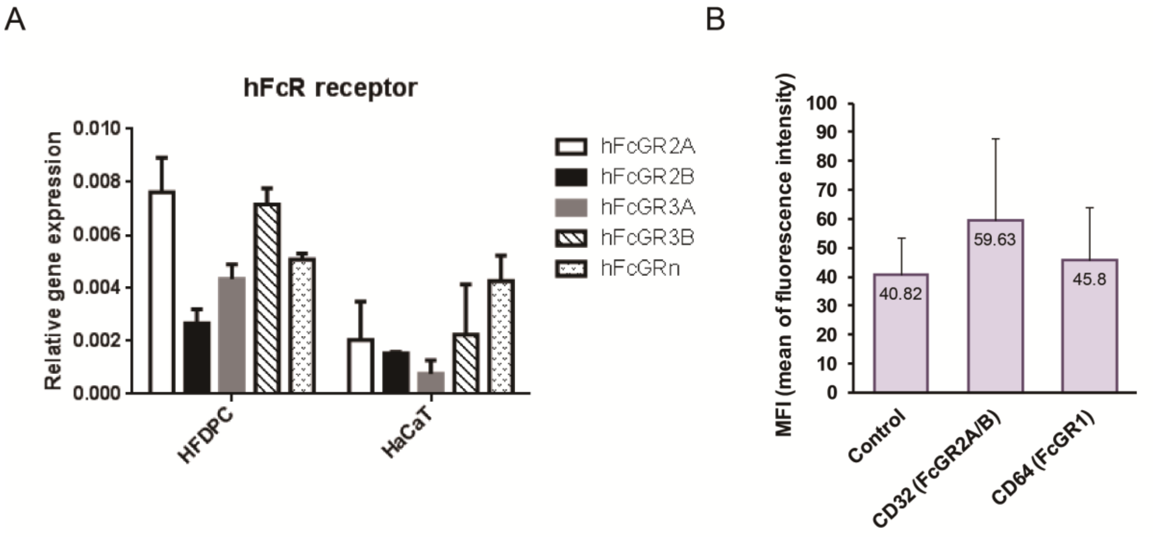

Figure S1. (A) The expression of human Fc gamma receptors in HFDPC. The mRNA expression of human Fc gamma receptor 2A/2B (hFcGR2A/2B, CD32), hFcγR3A/3B and neonatal Fc gamma R (hFcGRn, hFcγRn) were analyzed by RT-qPCR in HFDPCs and HaCaT cells (CLS, cell line service cryovial # 300493). The gene expression was normalized to GAPDH gene. (B) FACS analysis of hFcγR2A/2B9 (CD32) and hFcγR1 (CD64) on cell surface of HFDPCs. The antibody against CD32 and CD64 were from eBioscience.

Figure S2

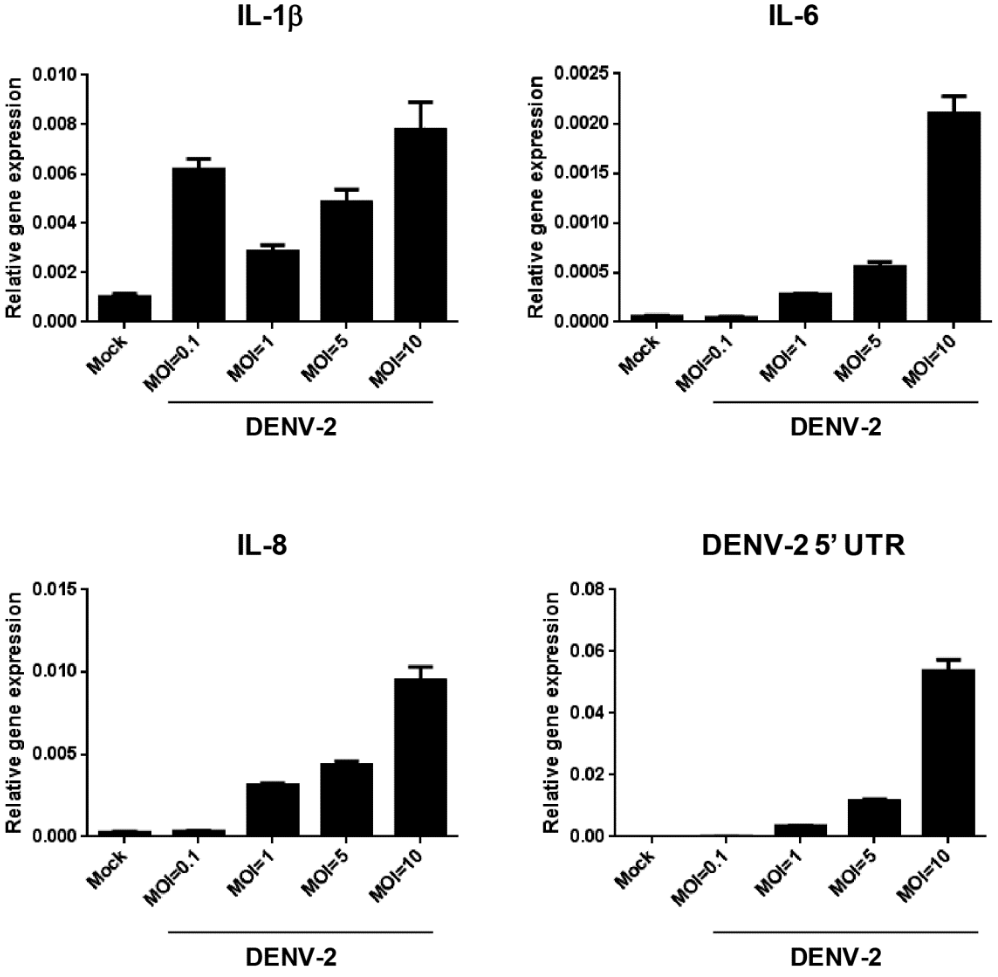

Figure S2. Induction of IL-1β, IL-6 and IL-8 expression and the DENV-2 5' untranslated region by various MOI (0.1-10) of DENV-2. Data are mean±SD from 3 independent tests.

Figure S3

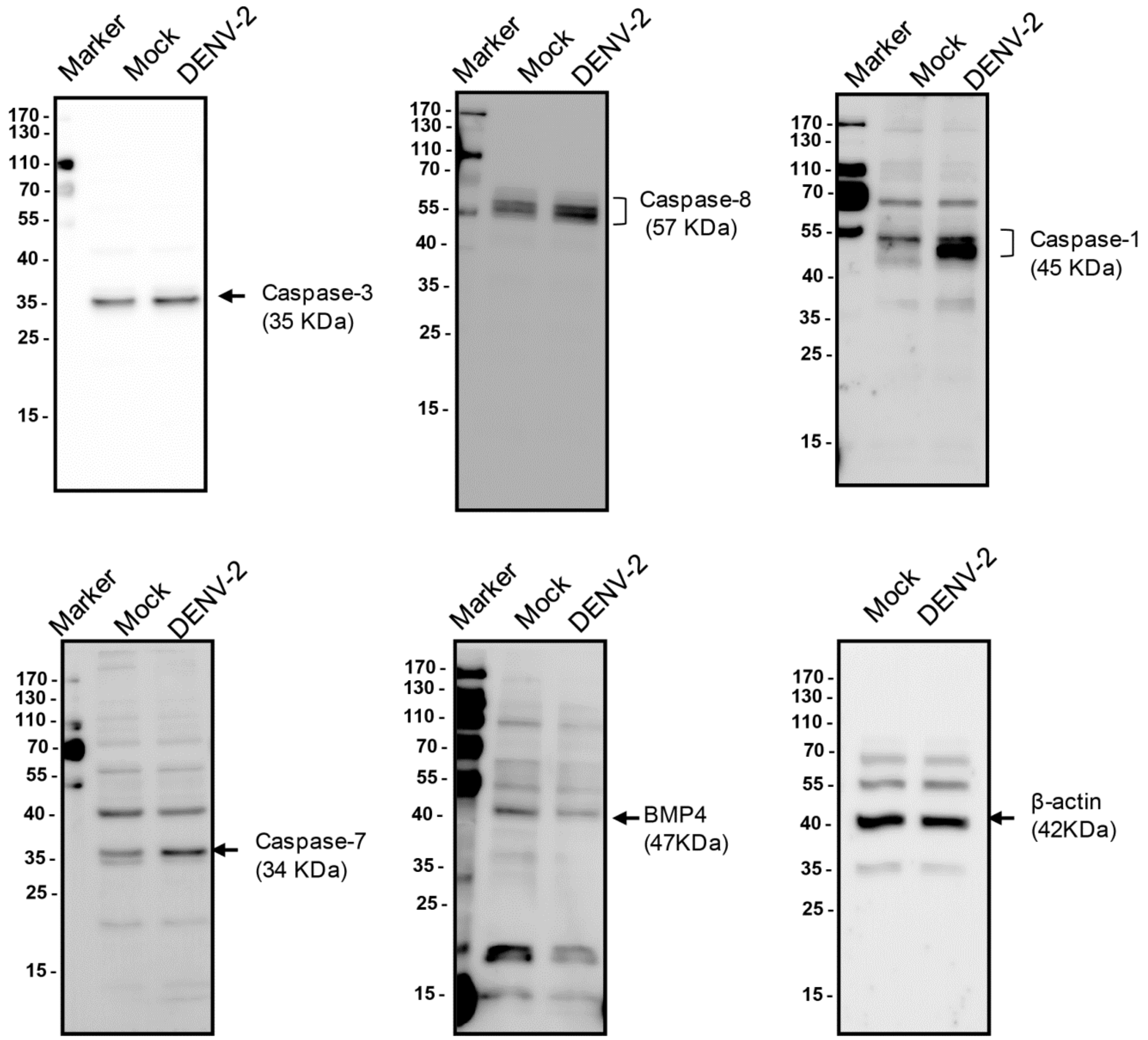

Figure S3. The full images of western blotting**.** The images showed the full immunoblotting membrane of cropped blots in Figure 5B.

Figure S4

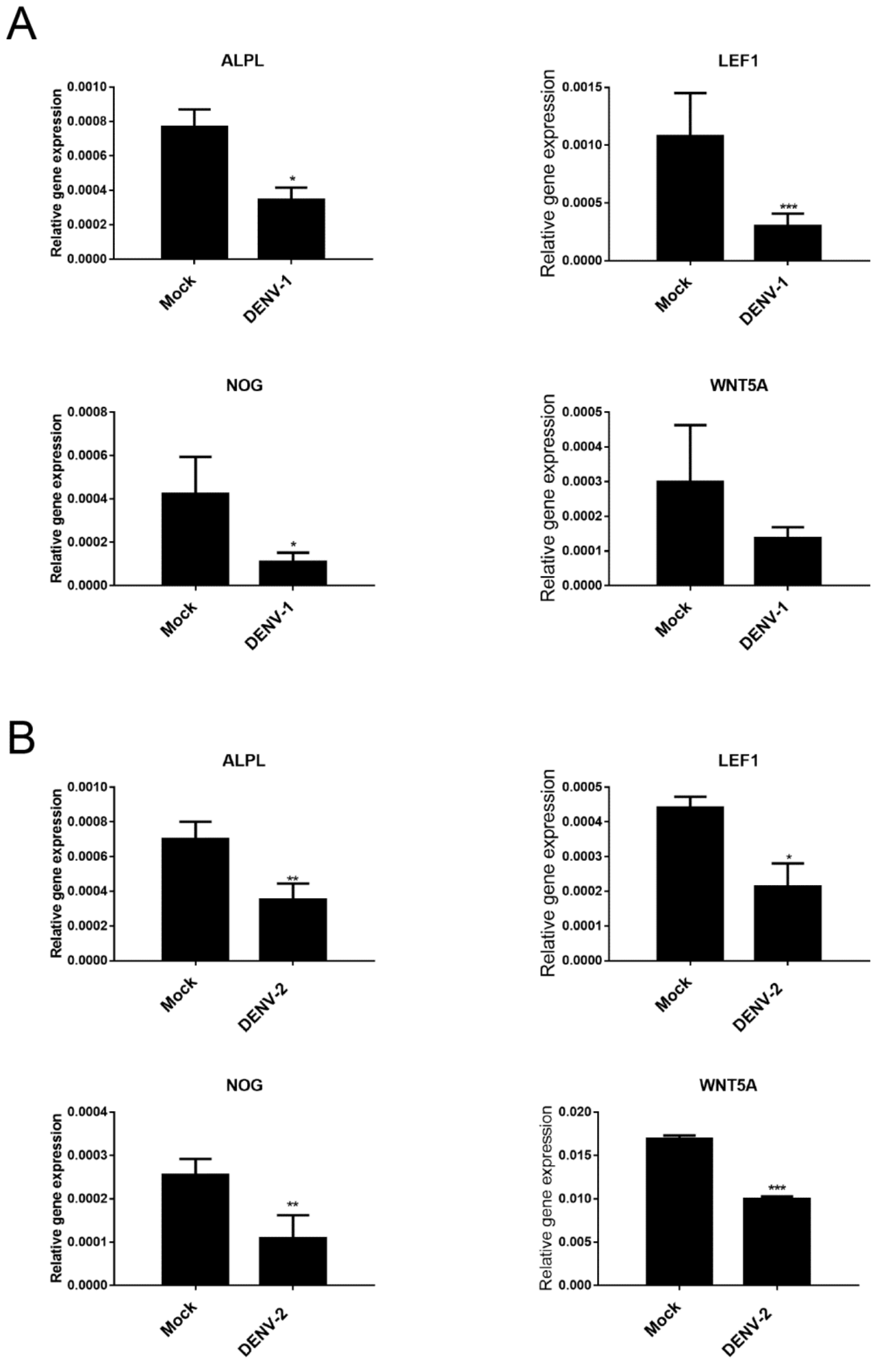

Figure S4. Reduction of ALPL, LEF1, NOG and WNT5A expression by DENV-1 (A) and DENV-2 (B) infection at MOI=10. Data are mean±SD. *, P<0.05 and **, P<0.01 vs Mock control.

Figure S5

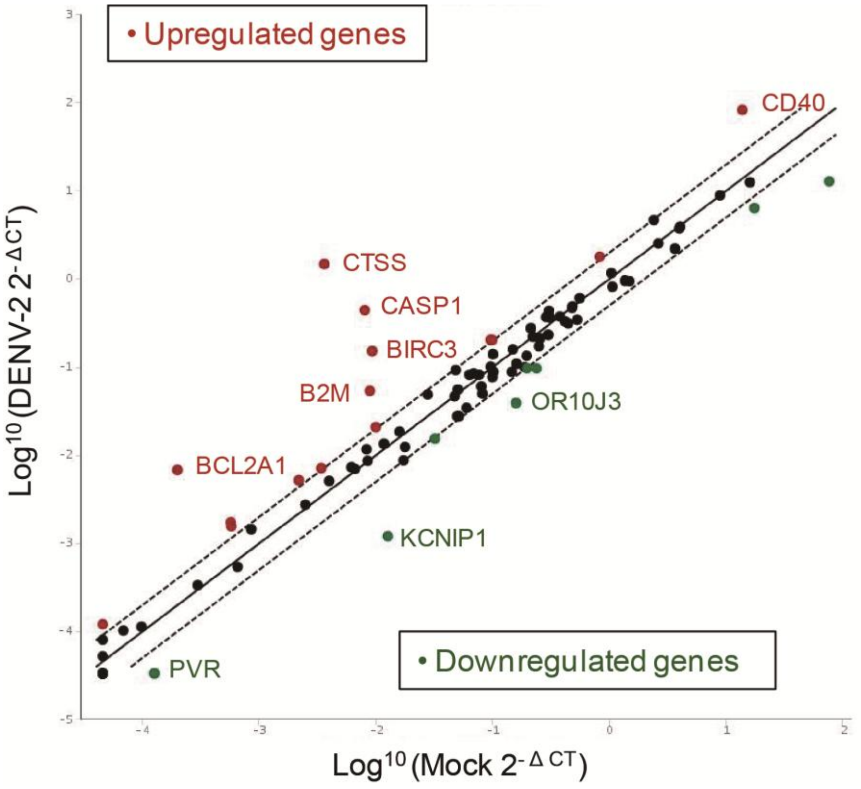

Figure S5. RT[2] profiler PCR of DENV-triggered cell-death signaling pathway. Overview of scatter plot on expression of 84 genes. Red dots are genes upregulated and green dots are genes downregulated in DENV-infected HFDPCs, as compared to control groups (Mock). The central line indicates unchanged gene expression; boundaries represents the two-fold regulation cut-off.
